# Ferroptosis, a Regulated Neuronal Cell Death Type After Intracerebral Hemorrhage

**DOI:** 10.3389/fncel.2020.591874

**Published:** 2020-11-16

**Authors:** Qinqin Bai, Jiachen Liu, Gaiqing Wang

**Affiliations:** ^1^Shanxi Medical University, Neurology, Taiyuan, China; ^2^Xiangya Medical College of Central South University, Clinical Medicine, Changsha, China; ^3^Department of Neurology, Sanya Central Hospital (HaiNan Third People’s Hospital), Sanya, China

**Keywords:** ferroptosis, intracerebral hemorrhage, lipid peroxidation, antioxidation, iron metabolism

## Abstract

Ferroptosis is a term that describes one form of regulated non-apoptotic cell death. It is triggered by the iron-dependent accumulation of lipid peroxides. Emerging evidence suggests a link between ferroptosis and the pathophysiological processes of neurological disorders, including stroke, degenerative diseases, neurotrauma, and cancer. Hemorrhagic stroke, also known as intracerebral hemorrhage (ICH), belongs to a devastating illness for its high level in morbidity and mortality. Currently, there are few established treatments and limited knowledge about the mechanisms of post-ICH neuronal death. The secondary brain damage after ICH is mainly attributed to oxidative stress and hemoglobin lysate, including iron, which leads to irreversible damage to neurons. Therefore, ferroptosis is becoming a common trend in research of neuronal death after ICH. Accumulative data suggest that the inhibition of ferroptosis may effectively prevent neuronal ferroptosis, thereby reducing secondary brain damage after ICH in animal models. Ferroptosis has a close relationship with oxidative damage and iron metabolism. This review reveals the pathological pathways and regulation mechanism of ferroptosis following ICH and then offers potential intervention strategies to mitigate neuron death and dysfunction after ICH.

## Introduction

Ferroptosis, a regulated non-apoptotic cell death, is characterized by overwhelming lipid peroxidation in an iron-dependent manner (Dixon et al., 2012; Stockwell et al., 2017). It was identified firstly in 2012 by handling tumor cells with the chemical probe erastin (Dixon et al., 2012). As described below, the regulation of ferroptotic death is dramatically modulated by lipid peroxidation, antioxidant system, and iron metabolism. Emerging evidence suggests that a link between ferroptosis and the pathophysiological processes of neurological disorders, including stroke, degenerative diseases, neurotrauma, and cancer (Li et al., 2020). Here, we will elaborate on neuronal ferroptosis after intracerebral hemorrhage (ICH).

ICH is an acute subtype of cerebral stroke and accounts for 80% of hemorrhagic stroke and 10–15% of all types of strokes (Donkor, 2018). Only six of 10 patients can survive 1 month after the onset of ICH (Li et al., 2018), and the poor outcomes after ICH result from complicated pathological processes that facilitate neuronal death. After ICH, Hb/heme/iron is recognized as one of the main contributors to delayed cerebral edema and irreversible damage to neurons and plays an essential role in lipid reactive oxygen species (ROS) production (Xiong et al., 2014). It was discovered that ferroptosis, iron-dependent cell death, exactly occurs after ICH and makes a contribution to the death of neurons, so the manipulation of ferroptosis may preserve neuronal cells exposed to specific oxidative conditions (Dixon et al., 2012; Li et al., 2017a; Wan et al., 2019). It has previously been observed that the inhibitor of ferroptosis reduced iron deposition and prevented neuronal death induced by hemoglobin (Hb; Li et al., 2017a). However, the specific agencies of neuronal ferroptosis after ICH are unclear. This review seeks to investigate the regulatory mechanisms of ferroptosis and how ferroptosis works in neuronal death after ICH. Based on this, potential intervention strategies to mitigate neuronal cell death and dysfunction after ICH are also summarized.

## The Mechanisms and Regulation of Ferroptosis

Essentially, ferroptosis is a form of programmed cell death induced by iron-dependent lipid peroxidation. Dysregulation of iron handling, increased of the labile redox-active iron, and increase of lipid peroxidation are viewed as possible pathogenic mechanisms of ferroptosis, so ferroptosis is related to lipid peroxidation, antioxidant system, and iron metabolism. Here we will elaborate on the mechanisms underlying ferroptosis and its regulatory systems.

### The Lipid Peroxidation Pathway in Ferroptosis

Lipid peroxidation refers to the process that oxygen combines with lipids to generate lipid hydroperoxides through the formation of peroxyl radicals, which is necessary for the execution of ferroptosis. It is confirmed that ferroptosis selective preferentially oxidizes specific polyunsaturated fatty acids (PUFAs) which contains phosphatidylethanolamine such as arachidonic acid (AA), leading to lipid peroxidation and ferroptosis (Song and Long, 2020). A recent study indicated that AA-OOH-PE induced ferroptosis (Kagan et al., 2017). In this process, the formation of AA-CoA is catalyzed by the acyl-CoA synthetase long-chain family 4 (ACSL4; Doll et al., 2017). Then, lysophosphatidylcholine acyltransferase 3 (LPCAT3) would esterify it to AA-PE (Dixon et al., 2015), which is oxidized into AA-OOH-PE by lipoxygenases (LOXs; Yang et al., 2016). When the content of AA-OOH-PE overwhelms the ability of the reduction system, ferroptosis will occur. Fatty acid desaturases can promote the formation of lipids containing PUFA, so it is a regulatory target for lipid peroxidation associated with ferroptosis.

### The Antioxidant System of Ferroptosis

Lipid peroxidation is the outcome of ferroptosis, so the antioxidant system plays an essential role in preventing ferroptosis. We will describe the antioxidant system that induces ferroptosis in two aspects, GSH and FSP1- CoQ10- NAD(P)H pathway.

#### GPX4 and GSH in Ferroptosis

**Figure 1:**
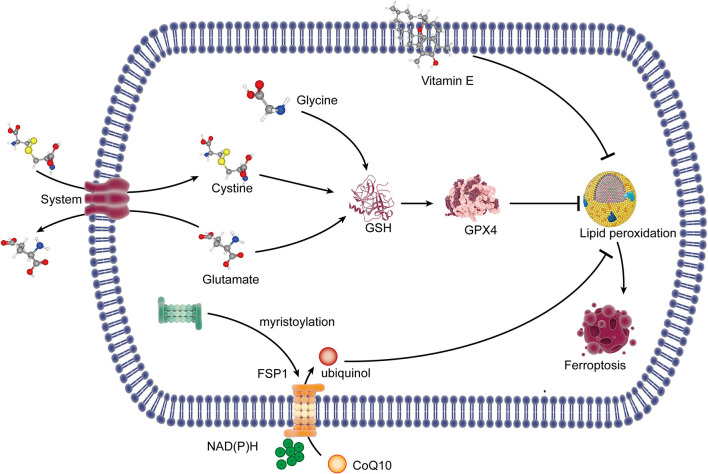
The lipid peroxidation pathway and the antioxidant system of ferroptosis. System xc^−^ exports glutamate out of the cell and imports cystine into the cell. Cystine in the cell is reduced to cysteine, which combined with glycine and glutamate for the synthesis of GSH. GSH is a synthetic substrate for glutathione peroxidase 4 (GPX4), which can resist lipid peroxidation and ferroptosis. FSP1 is transferred to the plasma membrane through myristoylation, where it mediates the reduction of CoQ10 to ubiquinol using NAD(P)H, which inhibits lipid peroxides. Vitamin E can inhibit lipid peroxidation with its radical-trapping activities. GSH, glutathione; FSP1, ferroptosis suppressor protein 1; CoQ10, coenzyme Q10; NAD(P)H, nicotinamide adenine dinucleotide phosphate.

Glutathione peroxidase 4 (GPX4), a selenium-dependent endogenous antioxidant enzyme, can complete the conversion of lipid peroxides to non-toxic lipids which will resist lipid peroxidation (Ursini et al., 1985) and then prevent ferroptotic death. Glutathione (GSH) is the synthetic substrate for GPX4 and is a cofactor for GPX4 to exert its antioxidant function (Feng and Stockwell, 2018), so it is currently regarded as the critical regulator of ferroptosis. This study suggests that GSH and selenium are necessary to maintain the operation of GPX4 and resist ferroptosis (Yan et al., 2020). GSH is synthesized by glycine, glutamate, and cysteine. Cysteine is transformed by intracellular cystine with a quick reduction reaction, and the transfer of cystine into the cell is promoted by System xc^−^, accompanied by glutamate out of the cell. System xc^−^, a heterodimeric cystine/glutamate antiporter, is made up of catalytic subunit solute carrier family 7 member 11 (SLC7A11) and solute carrier family 3 member 2 (SLC3A2). In cells where cysteine is obtained with the supply of cystine from system xc^−^, glutathione depletion is caused by the inhibition of the system xc^−^ (Dixon et al., 2014), so it is the most critical event of ferroptosis (Figure 1).

The inhibition of system xc^−^ can result in cysteine reduction, and the lack of GSH synthesis then reduced the antioxidant function of GPX4, which finally caused lipid peroxidation, leading to ferroptosis. The inhibitors of system xc leading to ferroptosis should rely entirely on the inhibition of cystine uptake. Erastin, piperazine erastin (PE), and imidazole ketone erastin (IKE) induce ferroptosis by the inhibition of system xc^−^, but PE and IKE have substantially improved potency (Dixon et al., 2012; Stockwell and Jiang, 2020). DPI2 and RSL5 may also be system xc^−^ inhibitors because of the similar effects to erastin, although the potential mechanism has not yet been reported (Stockwell and Jiang, 2020). Besides that, the multi-targeted kinase inhibitor sorafenib inhibits system xc^−^ function and can trigger ferroptosis, while a necrotic death can be induced when it comes to slightly higher concentrations (Dixon et al., 2014). Sulfasalazine is another kind of system xc^−^ inhibitor (Gout et al., 2001), but there is currently no reliable evidence that sulfasalazine can lead to ferroptosis. Furthermore, it is proven that glutamate and amino acid can inhibit system xc^−^ and promote ferroptosis in specific cellular contexts (Murphy et al., 1989). Lipid peroxidation occurs when the activity of GPX4 is inhibited. It suggested that GPX4 can be covalently inhibited by another inhibitor, named RSL3, which blocks the antioxidant system of GPX4 (Yang et al., 2014) and leads to ferroptosis. The transcription factor nuclear factor erythroid 2-related factor 2 (Nrf2) increases the resistance to ferroptosis by upregulating xCT, glutathione, and GPX4 (Chen et al., 2020). Selenium is proven to be an indispensable micronutrient for the function of GPX4, so when it was replaced by sulfur in GPX4, GPX4 fails in the activity of resisting overoxidation and inhibiting ferroptosis (Ingold et al., 2018; Song and Long, 2020).

#### FSP1-CoQ10-NAD(P)H Pathway in Ferroptosis

A recent study indicated that the ferroptosis suppressor protein 1 (FSP1)-coenzyme Q10 (CoQ10)-nicotinamide adenine dinucleotide phosphate [NAD(P)H] pathway existed as a stand-alone parallel system independent of GPX4 and GSH, which could also play an antioxidant role and inhibit ferroptosis (Doll et al., 2019). FSP1 is transferred to the plasma membrane through myristoylation, where it mediates the reduction of CoQ10 to ubiquinol using the reducing equivalents of NAD(P)H (Bersuker et al., 2019; Doll et al., 2019; Li and Li, 2020), and ubiquinol is a lipophilic radical-trapping antioxidant (RTA) that exerts an antioxidant effect to inhibit lipid peroxides (Li and Li, 2020). Vitamin E, a lipid-soluble antioxidant localized in the cell membrane, is also a RTA that can restrain lipid peroxidation through its radical-trapping activities (Matsushita et al., 2015; Bersuker et al., 2019; Figure 1).

### The Iron Metabolism-Related Pathway in Ferroptosis

Ferroptosis is iron-dependent cell death. Iron is a metal with redox activity and participates in the formation of free radicals and lipid peroxidation. Therefore, an increase of iron may promote ferroptotic death. Hydroperoxy lipids (L-OOH), the main enzymatic products of lipid peroxidation, are catalyzed by the iron centers of LOXs, and its decomposition products, oxidatively truncated electrophilic products, are yielded by Fe(II) from the labile iron pool, which triggers lipid peroxidation (Stoyanovsky et al., 2019; Bayir et al., 2020), eventually leading to ferroptosis. Therefore, iron metabolism can regulate ferroptosis.

**Figure 2:**
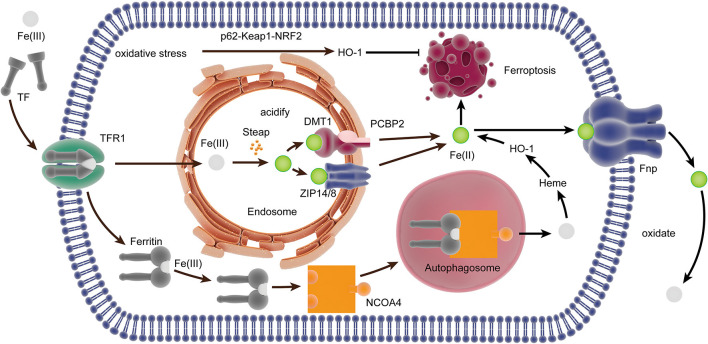
The iron metabolism-related pathway in ferroptosis. In the plasma, Fe(III) combined with TF to form TBI, which bound to TfR1 internalized by endocytosis. Iron is liberated from TF and reduced to Fe(II) in the endosome, which was transported into the cytosol by ZIP14/8 and divalent metal transporter 1 (DMT1) interacted by PCBP2. NCOA4 can directly deliver ferritin to the autophagosome, and it is degraded to Fe(III) for heme synthesis. Heme is degraded to Fe(II) by HO-1, which is transported out of the cell by Fpn. The expression of HO-1 can be upregulated by oxidative stress through the p62-Keap1-NRF2 pathway, thereby inducing ferroptosis. TF, transferrin; TBI, transferrin-bound iron; TfR, transferrin receptor; ZIP, ZRT/IRT-like protein; DM, divalent metal transporter; PCBP, poly rC binding protein; NCOA4, nuclear co-activator 4; Poly rC, binding protein; HO-1, heme oxygenase.

Under physiological conditions, Fe(II) can catalyze the transformation of hydrogen peroxide into a highly reactive intermediate substance, which attacks and oxidatively damages multiple cellular components, especially lipids containing PUFAs (Bayir et al., 2020). Almost all iron in the plasma is Fe(III) and combine with circulating transferrin (TF) to form transferrin-bound iron (TBI). Then, TBI is transported into the cell through endocytosis after combining with transferrin receptor 1 (TfR1) on the cell surface. Also, Fe(III) can be transported directly into cells without binding to Tf. Iron is liberated from TF and reduced to Fe(II) by the ferric reductases in the Steap family when the endosomal environment is acidified, and Fe(II) is transported into the cytosol across the membrane through ZRT/IRT-like protein (ZIP) 14 or 8 or divalent metal transporter 1 (DMT1; Ohgami et al., 2006; Stoyanovsky et al., 2019), and the poly rC binding-protein (PCBP) family, PCBP2, can combine iron and promote its transport into the cytosol by interacting with DMT1 (Yanatori et al., 2014). Ferritin transporter (Fpn), the only iron transmembrane export, can export Fe(II) out of the cells (Drakesmith et al., 2015). PCBP2 modulates the export of iron in the cells by delivering cytosolic iron to Fpn (Yanatori et al., 2016). Moreover, PCBP1 can bind iron and promote its loading onto client proteins like ferritin, which can be affected by PCBP2 (Stoyanovsky et al., 2019). Once Fe(II) is transported out of the cell, it is quickly oxidized to Fe(III) to be loaded onto TF. Under conditions of iron deficiency, nuclear co-activator 4 (NCOA4) can directly deliver ferritin to autophagosomes and facilitate its autophagic degradation (Mancias et al., 2014), which transferred back to the cytosol or the mitochondria for heme synthesis (Figure 2).

Heme oxygenase (HO)-1 has the dual effect of promoting and inhibiting ferroptosis by regulating iron. HO-1 mainly catalyzes the decomposition of heme into Fe (II), and the accumulation of Fe (II) has pro-oxidant activity and helps induce ferroptosis (Chiang et al., 2018). However, the free Fe (II) produced by HO-1 alone does not facilitate ferroptosis (Adedoyin et al., 2018). An earlier study has shown that the expression of HO-1 can be upregulated by oxidative stress through the p62-Keap1-NRF2 pathway, thereby inducing ferroptosis (Sun et al., 2016). Moreover, the increase of HO-1 will also affect intracellular iron distribution through enhanced heme degradation and ferritin synthesis (Lanceta et al., 2013). A study suggests that the activation of the medium level of HO-1 plays a role in cell protection, while the excessive activation of HO-1 plays a role in cytotoxicity because of the over-regulation of unstable Fe(II) (Chiang et al., 2018).

## The Underlying Mechanisms of Neuronal Ferroptosis After ICH

**Figure 3:**
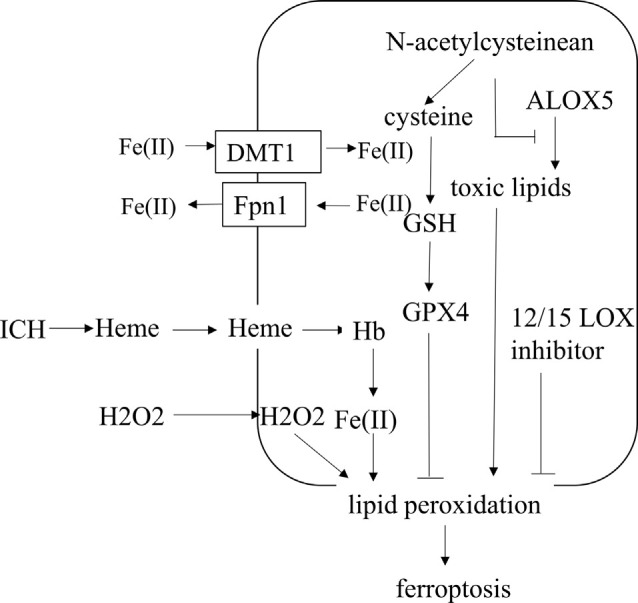
The regulatory mechanism of ferroptosis following intracerebral hemorrhage (ICH). After ICH, the heme incorporates into the plasma membrane and enhances lipid peroxidation by increasing the sensitivity to exogenous H_2_O_2_. N-acetylcysteinean can decrease toxic lipids produced by ALOX5 and increase the level of cysteine to prevent lipid peroxidation. Selenium, delivered into the brain, could increase the level of GPX4. Fe(II) through both Fpn1 input and DMT1 output increases after ICH. ALOX5, arachidonate-dependent arachidonate 5-lipoxygenase.

ICH is a fatal subtype of stroke with high mortality and morbidity because there are few established treatments and limited understanding of the type and related mechanisms of neuronal death after ICH. Various types of neuronal death after ICH, including apoptosis, pyroptosis, necrosis, ferroptosis, and autophagy, have been observed (Li et al., 2018; Zhao et al., 2018). Ferroptotic cells show shrunken mitochondria and increased membrane density in transmission electron microscopy, which is the difference between ferroptosis and other types of cell death (Dixon et al., 2012; Xie et al., 2016). In the ICH cell model and ICH-affected human brain tissue, the expression of iron death-related genes is upregulated (Chen et al., 2019). It has been shown that ferroptosis is present in the acute phase of ICH, and it also happens in neurons far from the center of the hematoma (Chen et al., 2019). After ICH, the iron released from Hb in the blood can produce a large number of ROS, which leads to oxidative stress in neuronal cells and secondary brain injury. The pharmacologic inhibition of ferroptosis is beneficial in animal models of ICH, revealing that ferroptotic inhibitors can be used as new agents for the treatment of ICH (Bartnikas et al., 2020). After ICH, mice treated with ferrostatin-1, a specific inhibitor of ferroptosis, could prevent the death of neurons by inhibiting lipid ROS production and reduce iron deposition induced by Hb, thereby exerting a long-term neuroprotective effect and improved neurological function (Li et al., 2017a; Zille et al., 2017; Chen et al., 2019). Here, we explore the underlying regulatory mechanism of ferroptosis following ICH (Figure 3).

### Lipid Peroxidation in Ferroptosis After ICH

A large amount of ROS can promote cell damage in the way of lipid peroxidation due to the disruption of the dynamic balance between the antioxidant system and ROS. Hb is the main component in blood, and Hb/heme/iron plays an indispensable role in the production of ROS and lipid ROS after ICH, which leads to secondary brain damage. After ICH, heme can incorporate into the plasma membrane and facilitate lipid peroxidation by increasing the sensitivity to the exogenous H_2_O_2_ (Robinson et al., 2009; Chen-Roetling et al., 2014). ICH-induced ROS can lead to cell damage through lipid peroxidation, including apoptosis and another parallel pathway, maybe ferroptosis (Qu et al., 2016). Oxygen-free radicals can enhance lipid peroxidation, which can cause the foundation of ferroptosis (Duan et al., 2016). Edaravone, a scavenger for oxygen free radicals, decreased cerebral edema and inhibited lipid peroxidation after intraventricular hemorrhage in rats (Chen et al., 2014). 12/15 LOX inhibitor, a lipid peroxidation inhibitor, reduces hemorrhagic transformation in warfarin-treated mice after experimental stroke, and there is a contribution of this inhibitor to intracerebral bleeding (Liu et al., 2017; Zheng et al., 2019). Ferroptosis is selective and preferentially oxidizes PUFAs. The content of PUFA glycerophospholipids is high in the brain, especially in neuronal membranes, so neurons are easily oxidized (Yan et al., 2020). However, there is no research on the connection between neuronal PUFA and ferroptosis after ICH, which requires further investigation. N-acetylcysteinean can neutralize toxic lipids produced by arachidonate-dependent arachidonate 5-lipoxygenase (ALOX5) to prevent lipid peroxidation and heme-induced neuronal ferroptosis after ICH (Zille et al., 2017). Therefore, inhibition of lipid peroxidation can prevent neurons from ferroptosis, which provides a therapeutic target for ICH.

### Antioxidant System for Ferroptosis After ICH

Redox balance is vital for the maintenance of brain health so that excessive oxidative reactions can result in neuronal death in the central nervous system. Oxidative stress and lipid peroxidation in neurons are critical for the occurrence of secondary brain injury after ICH, and ferroptosis is essentially lipid peroxidation damage in cells, so the ferroptosis-related antioxidant system can inhibit neuronal ferroptosis after ICH.

After ICH, ferroptosis is caused by a defect in the synthesis of GSH and the reduction of GPX4. It was reported that the systemic administration of N-acetylcysteinean, an approved cysteine prodrug, increased the levels of cellular cysteine and synthesis of GSH to inhibit neuronal ferroptosis after ICH (Zille et al., 2017). After ICH, GSH was significantly decreased (Wang et al., 2018), and GSH treatment in ICH mice could decrease brain edema and attenuate neural injury (Diao et al., 2020). It was shown that GPX4 protein levels were markedly reduced in neurons after ICH, the inhibition of GPX4 could exacerbate brain injury after ICH, and the upregulation of GPX4 could protect neurons from ferroptosis and ameliorate ICH-induced neuronal dysfunction in rats (Zhang et al., 2018). In addition, selenium delivered into the brain could promote the expression of antioxidant GPX4, inhibit neuronal ferroptotic death, and improve function in a hemorrhagic stroke model (Alim et al., 2019). The cystine/glutamate antiporter, system xc^−^, is the foundation of GSH production. The study indicated that glutaminolysis could contribute to the death of neurons after ICH *in vivo*, but it does not affect the toxicity induced by Hb *in vitro* (Li et al., 2017a). Moreover, it has been shown that inhibiting the activation of the oxide-metabolic driver activating transcription factor 4 can eliminate ferroptosis induced by glutamate analog and promote the recovery of brain function after ICH (Zille et al., 2019). Therefore, upregulation of GSH and GPX4 or increasing the functionality of the system xc^−^ may be a potential proposal to reduce brain damage caused by ICH.

### Iron Metabolism in Ferroptosis After ICH

**Figure 4:**
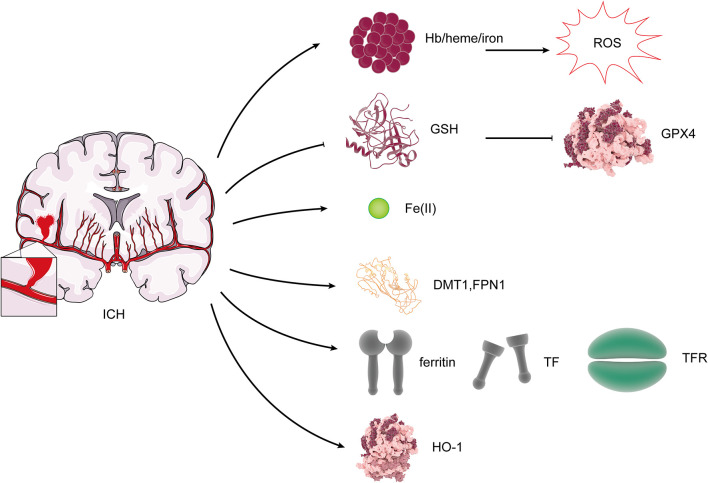
Changes of ferroptosis regulators after ICH. After ICH, Hb/heme/iron plays an indispensable role in the production of reactive oxygen species (ROS). The expression of GSH and GPX4 was reduced. DMT1, Fpn, ferritin, TF, TfR, and HO-1 levels were increased.

Iron, one of the essential elements, is vital for the function of many enzymes and the average survival of cells. However, iron can also result in cell damage due to its ability to catalyze the production of ROS (Wang et al., 2007). Ferroptosis, which is iron-dependent cell death, may occur through this process. The absence of iron response element binding protein 2 (IREB2) in neurons can lead to resistance in the toxicity of Hb, and it was shown that the expression of IREB2 mRNA was upregulated after ICH (Li et al., 2017a). Our previous study showed that DMT1 and Fpn1 increase in ICH rats, and there is a positive correlation between them and Fe(II) (Wang et al., 2015). Fpn1 upregulation is neuroprotective by facilitating Fe(II) export, but DMT1 has an adverse effect through enhancing iron import (Wang et al., 2016). The study indicated that the iron-handling proteins, including ferritin, TF, and TfR levels and HO-1, were significantly increased in the brain after ICH (Wu et al., 2003; Figure 4). Excessive iron is generated, causing brain damage after ICH. After ICH, the microglia and the macrophages were activated in the damaged area, which engulf Hb released from lysed red blood cells, degrade it, and release Fe(II) (Wan et al., 2019). Heme, the degradation product of Hb, can also act as a Fenton reagent, producing Fe(II) (Robinson et al., 2009). Then, Fe(II) is transported out of them and accumulates in neurons *via* the Tf–TfR system, which produces highly toxic hydroxyl radical, resulting in oxidative stress and the occurrence of lipid peroxidation (Wan et al., 2019). Ferroptosis, which is iron-dependent cell death, may occur through this process. After ICH, HO catalyzes the oxidation of heme to Fe(II). Two types of HO were found in the brain: HO-1 and HO-2. HO-1 is mainly expressed in astrocytes and microglia, while HO-2 is highly expressed by neurons (Robinson et al., 2009). A study suggests that ICH induces mostly the expression of HO-1, and HO-1 knockout mice exhibit smaller infarct volumes after ICH than wild-type mice (Wang and Doré, 2007), which shows that HO-1 is harmful to ICH. However, another study states that (-)-epicatechin, a brain-permeable flavanol, could reduce lesion volume and ameliorate neurologic deficits and neuronal degeneration *via* Nrf2 signaling, HO-1 induction, and brain iron deposition after ICH (Chang et al., 2014). After subarachnoid hemorrhage, microglial HO-1 is essential for virtually eliminating heme and attenuating neuronal cell death (Schallner et al., 2015), so HO-1 plays a dual role after ICH, which is similar to the effect of HO-1 on ferroptosis.

The efficacy of iron chelation therapy has been shown in the preclinical studies of ICH. Research showed the decrease of iron accumulation with iron chelators, including minocycline and VK-28, reduced ICH-related perihematomal iron accumulation, and improved neurological outcomes after experimental ICH (Li et al., 2017b; Dai et al., 2019). It is still not clear whether or not it inhibits ferroptosis.

## Summary

Ferroptosis is regulated nonapoptotic cell death caused by lipid peroxidation depending on the excessive production of ROS and the accumulation of intracellular iron, but its physiological mechanism has not been determined. When ferroptosis occurs, the morphological changes of mitochondria are most apparent, such as the contraction of mitochondria, the evolution of membrane potential, the reduction of mitochondrial cristae, electron-dense mass formation under ultrastructure, and rupture of mitochondria outer membrane (Song and Long, 2020). We find that it is mediated and regulated by lipid peroxidation, antioxidant system [including GPX4, GSH, system xc^−^, and FSP1-CoQ10- NAD(P)H pathway], and iron metabolism. Ferroptosis essentially results from an imbalance between oxidation and antioxidant systems.

ICH, a stroke subtype, is a disease that seriously affects the quality of life. It is characterized by a sudden rupture of cerebral blood vessels into surrounding brain tissue, which causes primary and secondary brain injury and irreversible damage to neurons. The physical compression of the hematoma causes primary damage, while the secondary damage is mainly caused by the lysis of red blood cells and the degradation of Hb, which then results in the accumulation of iron and the formation of ROS (Wagner et al., 2003; Aronowski and Zhao, 2011). More than one-third of patients with ICH will not survive, and most of the surviving patients have permanent disabilities (Bamford et al., 1990). The therapy for ICH remains elusive. Some patients undergo surgical hematoma evacuation, but there is no noticeable effect compared with conservative treatment (Bamford et al., 1990; Kuramatsu et al., 2019), so the therapeutic strategies for ICH remain elusive, and the study of secondary damage is of intense interest. After ICH, neuronal lipid peroxidation increases and the antioxidant GPX4 expression decreases, while DMT1, Fpn1, iron-treated protein, and HO-1 increase. Neuronal lipid peroxidation, the disorder of the antioxidant system, and iron metabolism are essential for secondary brain injury. Preclinical research suggests that the inhibition of ferroptosis can prevent neuronal ferroptosis after ICH. However, the detailed mechanism still needs further exploration to provide better treatment for patients with ICH.

In this review, we explained the underlying mechanism of neuronal ferroptosis. The generalizability of these results is subject to certain limitations, including the ferroptosis of astrocytes, oligodendrocytes, and microglia and their crosstalk. A study shows that ERK1/2, the vital marker of ferroptosis, is increased in astrocytes treated by heme (Regan et al., 2001). The reduced GSH in astrocytes was depleted through an inflammatory reaction induced by heme (Laird et al., 2008). Therefore, the ferroptosis of astrocytes may occur after ICH, which affects the function of neurons. GPX4 is localized to the nucleus of oligodendrocytes *in vivo*, and the inhibition of GPX4 induces ferroptosis in oligodendrocytes (Fan et al., 2021). Moreover, the upregulation of OH-1 in astrocytes and microglia affects the death of neurons. Therefore, there is a close connection between glia cell and neurons after ICH. The study indicated that the combined use of the inhibitors of different cell death has a better effect than only using one (Li et al., 2017a). In addition to ferroptosis, lipid peroxidation and increased ROS by oxidative stress after ICH can induce apoptosis *via* protein kinase C/protein kinase (CK2) pathway, NF-κB pathway, and cytochrome c (Hu et al., 2016). Superoxide is generated by excessive oxidative stress, resulting in the switch from apoptosis to necrosis (Duan et al., 2016). The inhibitors of necroptosis and ferroptosis each decrease neuronal death by greater than 80% and have similar windows for treatment *in vitro* (Zille et al., 2017). However, it remains to be studied how to induce different modes of cell death in a cell after ICH and what crosstalk occurs between them. Moreover, the temporal and the spatial characteristics of various cell deaths caused by ICH still require to be further explored. Solving the above problems may clarify the time window for therapy targeting different ways of cell death, which provides us with research directions and new targets for exploring the treatment of ICH.

In conclusion, neuronal ferroptosis may occur after ICH, and the inhibition of ferroptosis may prevent the ferroptotic death of neurons. Our review may bring insight on a possible option for ferroptosis-based ICH treatment in the future.

## Author Contributions

QB and JL drafted the manuscript and made the figures. GW made significant revisions to the manuscript. All authors contributed to the article and approved the submitted version.

## Conflict of Interest

The authors declare that the research was conducted in the absence of any commercial or financial relationships that could be construed as a potential conflict of interest.
